# Neutron Total Scattering Studies of Group II Titanates (ATiO_3_, A^2+^ = Mg, Ca, Sr, Ba)

**DOI:** 10.1038/s41598-020-60475-8

**Published:** 2020-02-28

**Authors:** Charles M. Culbertson, Alexander T. Flak, Michael Yatskin, Paul H.-Y. Cheong, David P. Cann, Michelle R. Dolgos

**Affiliations:** 1 Department of Chemistry, Oregon State University, Corvallis, Oregon 97331 USA; 2 Materials Science, School of Mechanical, Industrial & Manufacturing Engineering, Oregon State University, Corvallis, OR 97331 USA; 3 Department of Chemistry, University of Calgary, Calgary, Alberta Canada T2N 1N4, Canada

**Keywords:** Materials chemistry, Characterization and analytical techniques, Ceramics

## Abstract

Neutron total scattering measurements were conducted on MgTiO_3_, CaTiO_3_, SrTiO_3_, and BaTiO_3_ to simultaneously investigate the local and average structure of these materials. The local structures of MgTiO_3_, CaTiO_3_, and SrTiO_3_ were well modelled using the refined average structural models: trigonal *R*$\bar{3}$, orthorhombic *Pbnm*, and cubic *Pm*$\bar{3}$*m* respectively. However the local structure for BaTiO_3_, at both temperatures where the average structure is orthorhombic *Amm*2 and tetragonal *P*4*mm*, was best described by the rhombohedral *R*3*m* model. Only the *R*3*m* model was able to account for the observed displacement of titanium in the [111] direction. Furthermore, box-car type refinements were conducted. These refinements show that the coherence length of the rhombohedral distortion is around 10 Å, at larger *r*-ranges the local distortions become misaligned and average out to *Amm*2 and *P*4*mm*.

## Introduction

Alkaline earth metal (Group II) titanates of the form ATiO_3_ (A^2+^ = Mg, Ca, Sr, and Ba) are both technologically important and increasingly relevant for new material applications. These compounds are generally parent structures for complex materials that are used in a variety of applications. Magnesium titanate (MgTiO_3_) is a component in low-loss dielectric compositions for microwave applications, with a variety of uses including band-pass filters, communication antennas, direct broadcasting satellite and global positioning systems among others. Although an effective synthetic route has yet to be found for MgTiO_3_, limiting its current usage^1,2^. Calcium titanate (CaTiO_3_) is used in numerous applications, such as: optics, magnetics, electronics, and superconductors^3^. Most recently CaTiO_3_ has found renewed interest in hybrid organic-inorganic perovskites for solar cell applications^3^. Both strontium titanate (SrTiO_3_) and barium titanate (BaTiO_3_) are widely used in dielectric and ferroelectric applications^4^. The remaining group II elements beryllium and radium were not considered due to their toxicity and radioactivity, respectively.

Magnesium titanate crystallizes in the ilmenite structure which has the form ${{\rm{A}}}^{2+}{{\rm{B}}}^{4+}{X}_{3}^{2-}$. In general, the ilmenite structure is a layered derivative of the corundum structure where the cations are ordered into two nonequivalent octahedral A- and B-sites. The A and B sites are ordered along the hexagonal *c* direction in alternating layers of face shared A-X_6_ and B-X_6_ octahedra. The structure of MgTiO_3_ was first found to be *R*$\bar{3}$ by Posnjak and Barth in 1934^5^. A more recent study using neutron diffraction was conducted by Wechsler and Von Dreele in 1989^6^. This neutron diffraction study confirmed the average structure of MgTiO_3_ to be trigonal *R*$\bar{3}$ and found complete ordering of the Mg-O_6_ and Ti-O_6_ layers. The structure of MgTiO_3_ is stable over a wide range of temperatures (25–1025 °C) as no phase transitions have been reported^7^.

The rest of the materials (CaTiO_3_, SrTiO_3_, and BaTiO_3_) crystallize in the perovskite structure. The general perovskite structure is ABX_3_, where the A-site cations surrounded by 12 anions in cubo-octahedral coordination and the B-site cations are surrounded by 6 anions in octahedral coordination. The B-O_6_ octahedra form a network of corner sharing octahedra that surround the larger A-site cubo-octahedra. In the perovskite structure the B-site cations and anions are generally similar in size, whereas the A-site cations are relatively large in comparison. The relative size of the cations and anions can be used to predict the stability and distortions within the perovskite structure according to the Goldschmidt tolerance factor (*t*):

1 $$t=\frac{{r}_{A}+{r}_{O}}{\sqrt{2}({r}_{B}+{r}_{O})}$$

where *r*_A_ is the ionic radius of the A-site cation, *r*_B_ the radius B-site cation, and *r*_O_ the radius of oxygen. The value of the tolerance factor *t* can be used to predict the structure, for *t* < 0.71 the structure is generally not a perovskite, for *t* = 0.71–0.9 orthorhombic/rhombohedral symmetry is preferred, for *t* = 0.9–1 cubic is stable, and for *t* > 1 hexagonal or tetragonal distortions are preferred^8^. It should be noted that these tolerance factor (*t*) ranges are guidelines based on observations.

Most materials with the perovskite structure are not cubic, mainly due to cation displacements and octahedral rotations. The archetypical perovskite CaTiO_3_ is orthorhombic *Pbnm* (*t* = 0.97) from room temperature until 1375 K. At 1375 K the structure begins to undergo a phase transition to tetragonal *I*4/*mcm* and becomes single-phase tetragonal at 1425 K. The final structural transition from tetragonal to cubic *Pm*$\bar{3}$*m* occurs at 1525 K^9^.

At low temperatures the average structure of SrTiO_3_ is tetragonal *I*4/*mcm*, and around 100 K the structure transitions to the room temperature phase cubic *Pm*$\bar{3}$*m* (*t* = 1.00)^10^. It has been reported that a lower temperature (35–55 K) orthorhombic phase exists^11^. Although, a pair distribution function study of SrTiO_3_ at 5 and 293 K found only cubic symmetry present for the local structure^12^.

The average structure of BaTiO_3_ (*t* = 1.06) has been reported to undergo several phase transitions. Below 190 K, the structure is rhombohedral *R*3*m*. From 190 to 280 K the structure is orthorhombic *Amm*2, from 280 to 360 K the structure is tetragonal *P*4*mm*, and the structure finally becomes cubic *Pm*$\bar{3}$*m* above 360 K^13,14^. These reported structures are all related to the undistorted cubic phase and characterized by the direction of the B-site titanium atoms (*R*3*m* – [111], *Amm*2 – [011], *P*4*mm* – [001]). In general, these materials undergo displacive phase transitions, where the structure changes as atoms move due to increased thermal energy. Another type of phase transition is an order-disorder transition, where the structure of a material changes due to a change in the degree of positional or orientational ordering/disordering.

In 1968, an order-disorder model was proposed by Comes *et al*. for BaTiO_3_ based on the diffuse scattering from single crystals^15^. In this model, only the rhombohedral phase was considered ordered and the orthorhombic, tetragonal or cubic phases observed were the result of an averaging of titanium atom displacements in disordered rhombohedral directions^15^. Since then, many studies have been conducted on BaTiO_3_ using a variety of methods and techniques to explain the complex local and average structures. The methods and summarized results of these studies are listed in Table 1. The most recent study from Senn *et al*.^16^ describes the order-disorder behavior observed in BaTiO_3_ as emanating from local titanium displacements along the <111> directions associated with rhombohedral *R*3*m* symmetry. At low temperatures, these rhombohedral displacements are all aligned (along the [111] direction), but as the temperature increases they become disordered (i.e. [111] and [$\bar{1}11$]) to a vector average associated with orthorhombic *Amm*2 symmetry ([011] direction). At higher temperatures, the Ti displacements disorder along four possible directions ([111], [$\bar{1}11$], [$1\bar{1}1$] and [$\bar{1}\bar{1}1$]) to a vector average associated with tetragonal *P*4*mm* symmetry ([001] direction). The structure appears cubic *Pm*$\bar{3}$*m* at higher temperatures when the local rhombohedral displacements are completely disordered relative to one-another (eight possible displacement directions so that the net Ti displacement is localized in the center of the oxygen octahedron).

**Table 1:** Summary of literature studies on the structure of barium titanate.

Methodology	Conclusion	Date	Reference
X-ray Diffuse Scattering of Single Crystals	The rhombohedral phase is ordered, the other phases are partially ordered. The diffraction results are average structures	1968	Comes *et al.*^15^
Neutron Powder Diffraction	The structure was well fit with the average structure models and the anisotropic thermal parameters do not support the order-disorder model	1993	Kwei *et al.*^14^
Neutron Total Scattering	Local rhombohedral symmetry was found in rhombohedral and orthorhombic phases, but the average structure described the tetragonal and cubic phases well	1995	Kwei *et al.*^34^
Extended X-ray Absorption Fine Structure (EXAFS) and X-ray Absorption Near Edge Structure (XANES)	The local structure is rhombohedrally distorted at all temperatures, the average structures observed were explained by the disordering of domains	1998	Ravel *et al.*^35^
Nuclear Magnetic Resonance of Single Crystal	Satellite peaks were observed in the cubic phase that were attributed to tetragonal symmetry	2003	Zalar *et al.*^36^
Synchrotron Total Scattering of Nanocrystals	Tetragonal local symmetry with cubic average symmetry	2006	Petkov *et al*.^17^
RMC Modeling of Neutron PDF, EXAFS, and Diffuse Scattering	Tetragonal phase shows four displacements and cubic phase shows eight displacement directions for titanium atoms	2014	Levin *et al.*^37^
RMC Modeling of Neutron PDF	Local rhombohedral titanium distortions are correlated and lead to experimentally observed average structures	2016	Senn *et al.*^16^

Additionally, the local structures of nanocrystalline Ba_*x*_Sr_1–*x*_TiO_3_ (*x* = 1, 0.5, 0) have also been investigated by synchrotron total scattering. For nanocrystalline SrTiO_3_ the local and average structure were found to be cubic. However for nanocrystalline BaTiO_3_ the local structure was found to possess tetragonal distortions in the 10–15 Å regime, whereas the average structure of the nanocrystals was best described as cubic^17^.

In the present work, to provide insight into the local and average structure of these materials, neutron total scattering measurements of bulk ceramics were conducted. The advantage of collecting neutron total scattering is two-fold. Firstly, using total scattering one can study both the average and local structures of a material simultaneously. The process of simultaneous data collection is important, as a single model must be able to explain the apparent discrepancy between both coherent data sets. The second key advantage is intrinsic to neutron scattering, where neutron scattering is not dependent on the elemental number (Z) as is the case for X-ray scattering. In particular, oxygen ions scatter neutrons strongly (oxygen coh b = 5.80 fm) and titanium ions have a negative scattering lengths (titanium coh b = −3.438 fm) which provide better signal and contrast respectively for accurate refinements^18^. The advantages of neutron total scattering can be seen in the work of Page *et al*.^19^ who employed the technique on niobium doped SrTiO_3_ (SrTi_0.875_Nb_0.125_O_3_) and BaTiO_3_ (BaTi_0.875_Nb_0.125_O_3_). At room temperature the local structure of niobium doped SrTiO_3_ remains cubic like the parent structure of SrTiO_3_. For niobium doped BaTiO_3_, the local structure appears to possess rhombohedral symmetry^19^.

The purpose of this work is to provide the community with structural refinements on high resolution neutron total scattering measurements to clearly define both the local and average structure of the accessible group II titanates. In particular, the focus of this work is to present the refined structures of MgTiO_3_, CaTiO_3_, and SrTiO_3_, to serve as a reference point to understand the structural details in BaTiO_3_. Furthermore, this work seeks to provide a summarization of the literature studies on BaTiO_3_ to contextualize these nuances. To accomplish these goals, phase pure samples were synthesized, and neutron total scattering data were collected at both 225 and 290 K (particularly to show the phase sequence of BaTiO_3_) for all compositions.

## Results

### Magnesium Titanate

As discussed in the introduction, MgTiO_3_ crystallizes in the ilmenite structure with trigonal *R*$\bar{3}$ symmetry at room temperature. In the ilmenite structure both magnesium and titanium are in 6-coordinate octahedral oxygen environments^6^.

**Figure 1:**
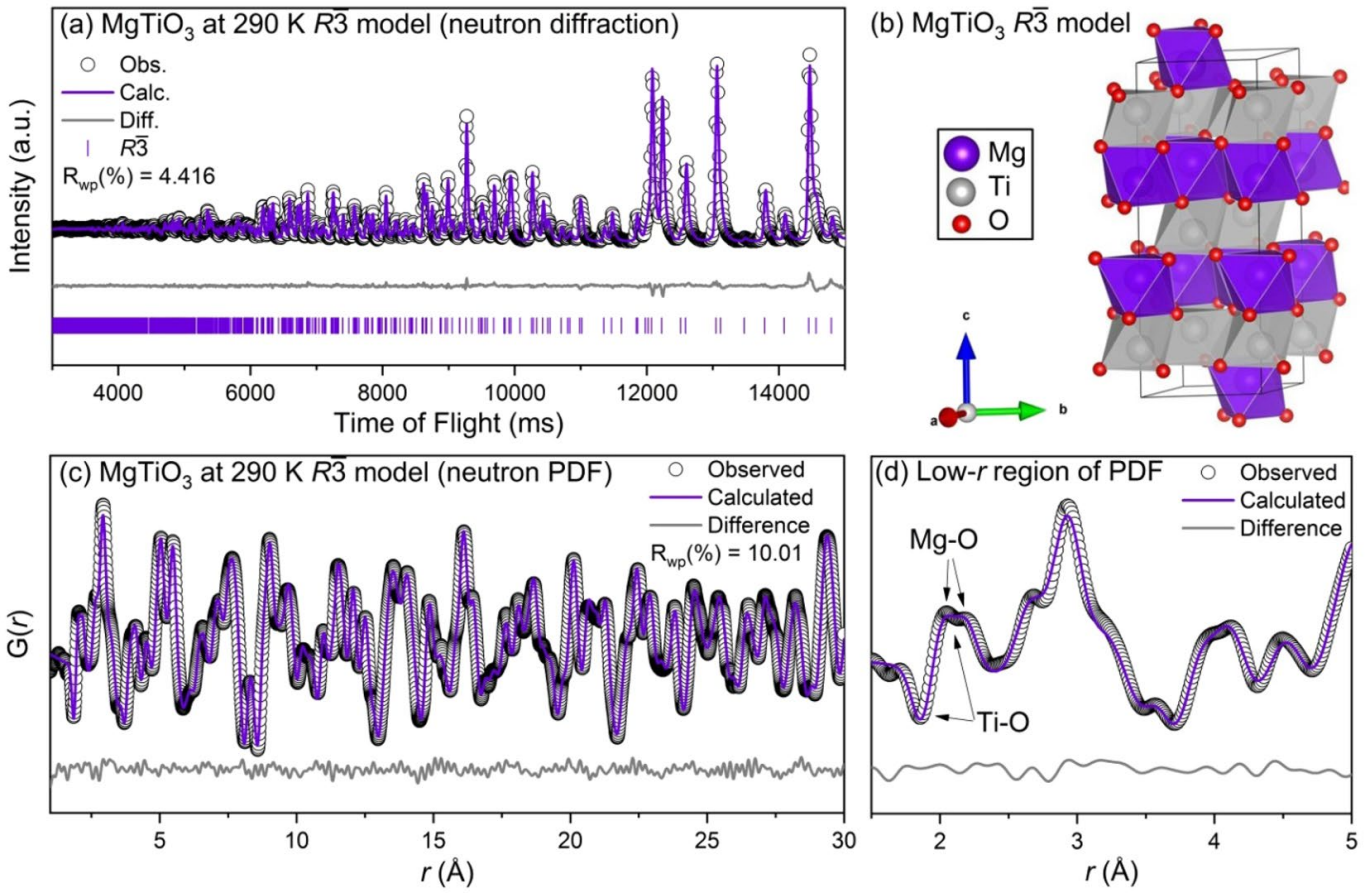
(**a**) Rietveld refinement of neutron diffraction, (**b**) refined model, (**c**) small-box modeling of neutron PDF, and (**d**) zoom-in of neutron PDF for MgTiO_3_ at 290 K with the *R*$\bar{3}$ space group. Data (identified by black circles) and refined models (continuous lines) are shown, along with the difference pattern and *hkl* indices below (diffraction data only).

From the initial X-ray diffractograms there was a minor MgTi_2_O_5_ phase observed in the MgTiO_3_ sample (shown in Supplemental Fig. S1). However this phase was not observed in the neutron total scattering data. The results of the diffraction and PDF refinements of MgTiO_3_ at 290 K with the *R*$\bar{3}$ space group in the equivalent hexagonal setting (*a* = *b*, α = β = 90°, γ = 120°) are shown in Fig. 1. The tabulated results for the refinement at 290 K and the complete results for the refinement at 225 K are included in the supplemental information. The diffraction refinement converged on a fit criterion, R_wp_ = 4.146%, with all reflections well modelled. The crystal structure of the refined structure is shown in Fig. 1(b), with magnesium shown as purple, titanium as grey, and oxygen as red spheres. The neutron PDF, shown in Fig. 1(c), was also well fit with the *R*$\bar{3}$ space group, R_wp_ = 10.012%. The titanium- and magnesium-oxygen bonds for the first coordination sphere are labelled in the low-*r* region of the PDF (Fig. 1(d)). Both titanium and magnesium are displaced in their respective oxygen octahedral, resulting in three short bonds at 1.8656 Å for titanium and at 2.0497 Å for magnesium and three long bonds at 2.0940 Å for titanium and 2.1660 Å for magnesium. Although there are A- and B-site displacements in the MgTiO_3_ structure, the *R*$\bar{3}$ space group is centrosymmetric and thus not a ferroelectric phase.

The negative scattering length of titanium mentioned previously manifests in negative peaks (or wells) for titanium correlations in the PDF. The diffraction and PDF refinements were conducted independently, but converged on the same model with slight discrepancies. Overall, both the long range average and short range local structure refinements clearly support literature reports describing the ilmenite structure for MgTiO_3_ at these temperatures^6^.

### Calcium titanate

At room temperature CaTiO_3_ crystallizes in the perovskite structure with *Pbnm* (or the equivalent *Pnma* and *Pcmn*) symmetry^20^. As discussed in the introduction, the perovskite structure is a network of corner shared B-site octahedra titanium atoms surrounding the 12-coordinate cubo-octahedral A-site calcium ions.

**Figure 2:**
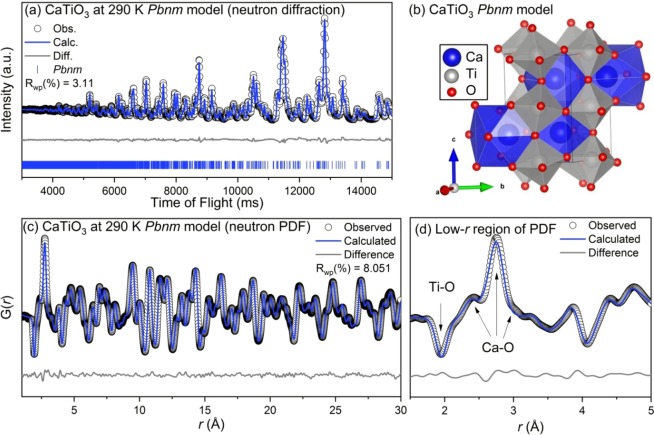
(**a**) Rietveld refinement of neutron diffraction, (**b**) refined model, (**c**) small-box modeling of neutron PDF, and (**d**) zoom-in of neutron PDF for CaTiO_3_ at 290 K with the *Pbnm* space group. Data (black circle’s) and refined models (continuous lines) are shown, along with the difference pattern and *hkl* markers below (diffraction data only).

The results of the neutron diffraction, R_wp_ = 3.11%, and neutron pair distribution function data, R_wp_ = 8.05%, for CaTiO_3_ at 290 K are shown in Fig. 2(a–d) respectively. The refinements for CaTiO_3_ at 225 K are included in the supplementary information. Both independent refinements at 290 K converged on the same *Pbnm* model where *a* = 5.38, *b* = 5.44, and *c* = 7.64 Å. Titanium is displaced towards an edge of the Ti-O_6_ octahedra, yielding two bonds at 1.9514, two at 1.9530, and two at 1.9643 Å. The calcium-oxygen cubo-octahedra is more complex in the refined model, with 8 unique bond distances ranging from 2.3379 to 3.2311 Å. The major peaks that result from these bonds are labelled in the low-r region of the PDF (Fig. 2(d)). Similarly to the results for MgTiO_3_, the average and local structure of CaTiO_3_ are well described at 290 K with the *Pbnm* space group as has been reported in the literature^20^. The *Pbnm* space group is centrosymmetric and thus CaTiO_3_ does not display ferroelectric properties.

### Strontium titanate

Across a large temperature regime SrTiO_3_ crystallizes in the perovskite structure with cubic *Pm*$\bar{3}$*m* symmetry^11^.

**Figure 3:**
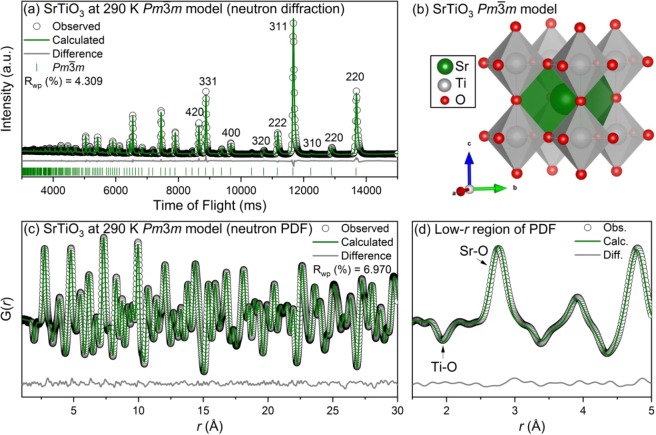
(**a**) Rietveld refinement of neutron diffraction, (**b**) refined model, (**c**) small-box modeling of neutron PDF, and (**d**) zoom-in of neutron PDF for SrTiO_3_ at 290 K with the *Pm*$\bar{3}$*m* space group. Data (black circles) and refined models (continuous lines) are shown, along with the difference pattern and *hkl* markers below (diffraction data only).

At 290 K the diffraction, R_wp_ = 4.309%, and PDF, R_wp_ = 6.970%, refinements results are shown in Fig. 3(a–d) respectively. The results of the refinement at 225 K are similar to those at 290 K and are included in the supplementary information. Due to the cubic symmetry of SrTiO_3_, the diffraction data shows only a few sharp peaks of which the 420 through 220 reflections are labelled. All of the observed reflections were well fit by the model, with no additional reflections observed. There was some observed peak asymmetry which is characteristic of the NOMAD beamline and spallation sources more generally. Similarly, all of the peaks in the PDF data (Fig. 3(c,d)) were well modelled. The titanium-oxygen and strontium-oxygen bonds are labelled in Fig. 3(d). The width of the single Ti-O peak is a result of the thermal disorder present even though all six Ti-O bonds are equivalent with a bond distance of 1.9534 Å. Similarly, strontium is positioned at the center of the Sr-O_12_ cubo-octahedra, with twelve Sr-O bonds at 2.7625 Å. The refined models for the average and local structure of SrTiO_3_ are consistent with what has been reported in the literature^11^.

### Barium titanate

The structure of BaTiO_3_ is much more complicated than the other titanates in its group, although it still crystallizes in the perovskite structure. The reported phase sequence for BaTiO_3_, from low to high temperature, is *R*3*m* to *Amm*2 to *P*4*mm* to *Pm*$\bar{3}$*m*. In this study data was collected at 225 K in the orthorhombic *Amm*2 region, and at 290 K in the tetragonal *P*4*mm* region^14^.

**Figure 4:**
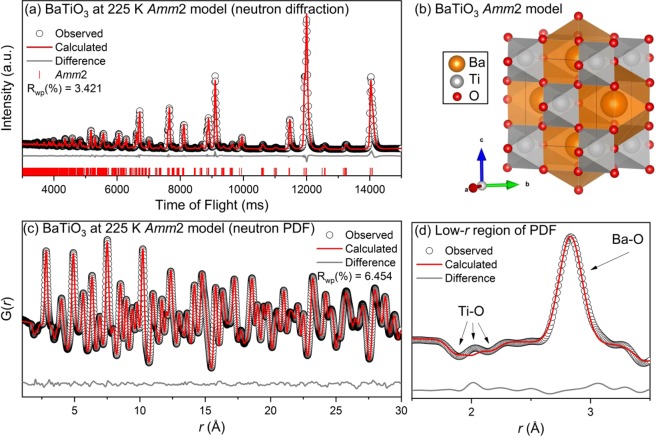
(**a**) Rietveld refinement of neutron diffraction, (**b**) refined model, (**c**) small-box modeling of neutron PDF, and (**d**) zoom-in of neutron PDF for BaTiO_3_ at 225 K with the *Amm2* space group. Data (black circles) and refined models (continuous lines) are shown, along with the difference pattern and *hkl* markers below (diffraction data only).

The results of the refinements for BaTiO_3_ at 225 K are shown in Fig. 4. For the Rietveld refinement, as expected, the *Amm2* model fit all of the observed reflections well, with R_wp_ = 3.421%. At first glance the PDF refinement seems equally successful, R_wp_ = 6.454%, however upon closer inspection it becomes clear that the *Amm*2 space group does not adequately model the first two Ti-O bonds at ~2 Å (Fig. 4(d)). The orthorhombic *Amm2* symmetry, which corresponds to a titanium displacement in the [011] direction, would result in a broad set of three shallow peaks in the PDF data for the Ti-O correlations (2 short bonds, 2 medium bonds, and 2 longer bonds) – which is not observed in the experimental data. In the data there are two equivalent negative peaks at ~1.9 and ~2.1 Å.

**Figure 5:**
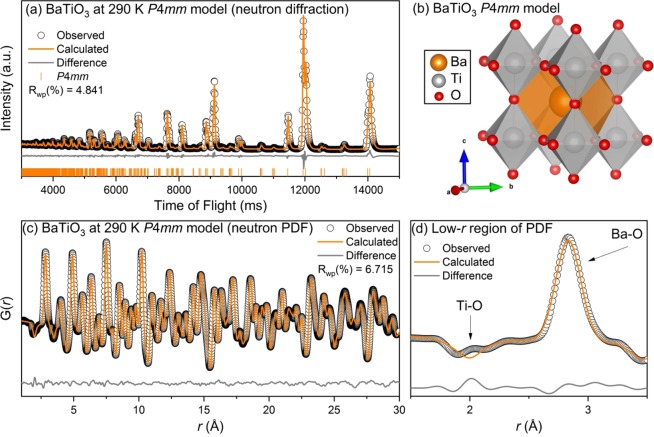
(**a**) Rietveld refinement of neutron diffraction, (**b**) refined model, (**c**) small-box modeling of neutron PDF, and (**d**) zoom-in of neutron PDF for BaTiO_3_ at 290 K with the *P*4*mm* space group. Data (black circles) and refined models (continuous lines) are shown, along with the difference pattern and *hkl* markers below (diffraction data only).

Similarly, the results for the refinements of BaTiO_3_ at 290 K in the tetragonal *P*4*mm* space group are shown in Fig. 5. The refinement of the diffraction data, Fig. 5(a), yielded a good fit where all of the observed reflections were modeled. The diffraction data showed peak splitting in the (220), (310), (311), (400), and (420) pseudocubic reflections, which are indicative of a tetragonal distortion in the [001] direction. As was the case with the refinement at 225 K, the *P*4*mm* model at 290 K does not adequately model the two Ti-O peaks in the PDF data. A [001] tetragonal titanium distortion would result in three Ti-O peaks (1 short bond, 4 medium bonds, 1 long bond), which was not observed.

**Figure 6:**
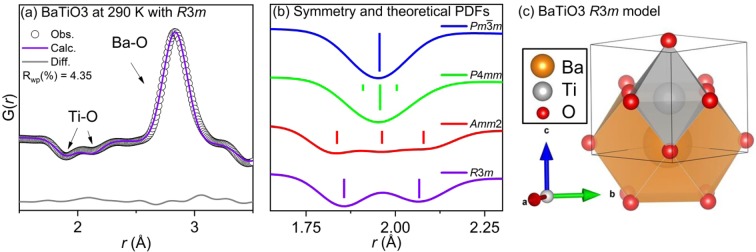
Small-box modeling of local structure (**a**) for BaTiO_3_ at 290 K using the *R*3*m* space group. Data (black circles) and refined model (continuous line) are shown with the difference pattern below. Correlation labels are provided for clarity within 1 unit cell. Calculated PDF (**b**) for *Pm*$\bar{3}$*m*, *P*4*mm*, *Amm*2, and *R*3*m* models with bond distances shown as tick lines. (**c**) Refined local structure *R*3*m* model.

To account for the two Ti-O peaks around 1.9 and 2.1 Å that were poorly fit by both the *Amm2* space group at 225 K and *P*4*mm* space group at 290 K, further refinements were conducted using each of the reported space groups for BaTiO_3_ (*R*3*m*, *Amm2*, *P*4*mm*, *Pm*$\bar{3}$*m*). The first two Ti-O correlations could only be modelled with the *R*3*m* space group. The results of the low-*r* region refinement at 290 K are shown in Fig. 6(a). The refinement at 225 K yielded an analogous fit and the tabulated results at both 225 K and 290 K are presented in the supplementary information. The bond structure and theoretical neutron PDFs of the reported average structures of BaTiO_3_ are shown in Fig. 6(b). The refined structure is shown visually in Fig. 6(c). The double peaks observed are characteristic of a rhombohedral distortion in the [111] direction, as the titanium atoms are displaced toward the face of the Ti-O_6_ octahedra. The face-displaced titanium octahedra results in 3 short bonds (~1.9 Å) and 3 longer bonds (~2.1 Å).

In the literature, there are two distinct methods that have been used to model local deviations from an average structure with small box models. The first method, henceforth referred to as the variable range refinement method, involves including an adjustable range of data (*x*-range) for each refinement from a fixed starting point (typically ~1 Å). For example, Smith *et al*. investigated BaTiO_3_ nanoparticles and refined the PDF data set from 1 to 28 Å, with varying refinement lengths (ranging from 1–8, 1–12, 1–16… 1–28 Å, etc.)^21^. The second method, known as the box-car method, involves taking fixed-length boxes and performing sequential refinements from low to high *r*-ranges. For example, in the study of BaTiO_3_-Bi(Zn_1/2_Ti_1/2_)O_3_ by Usher *et al*., a fixed refinement range of 10 Å was used to refine the structure from 1 to 80 Å (over proscribed ranges of 1–10, 5–15, … 70–80 Å, etc.)^22^. Both methods were utilized in this study to provide a more complete analysis.

**Figure 7:**
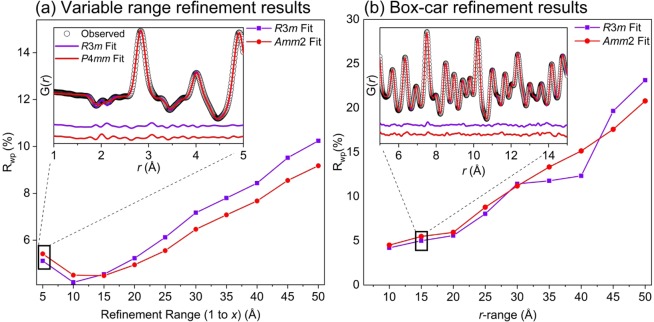
Results (R_wp_) of the small-box analysis using the (**a**) variable range refinement method (refinement range shown as *x*-axis, i.e. *x* = 5 corresponds to 1–5 refinement, *x* = 10 to 1–10, … *x* = 50 to 1–50 Å, etc.) and (**b**) box-car refinement method (r-range shown on the x-axis corresponds to the high-r edge of box, i.e. *x* = 10 corresponds to the 0–10 Å box, *x* = 15 to the 5–15 Å box, …, *x* = 50, the 40–50 Å box, etc.) for neutron PDF data of BaTiO_3_ at 225 K using the *R*3*m* (violet squares) and *Amm*2 (red circles) space groups. The insets for each method show an example of one refinement distance or box.

**Figure 8:**
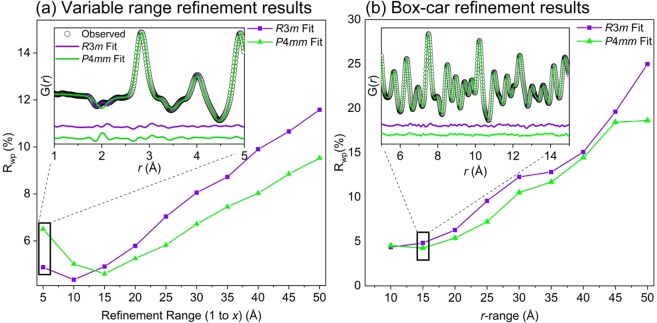
Results (R_wp_) of the small-box analysis using the (**a**) variable range refinement method and (**b**) box-car refinement method for neutron PDF data of BaTiO_3_ at 290 K using the *R*3*m* (violet squares) and *P*4*mm* (green triangles) space groups. The insets for each method show an example of one refinement distance or box.

The results of the variable range refinements and box-car refinements are shown in Figs. 7 and 8 for BaTiO_3_ at 225 and 290 K, respectively. The calculated fit criterion (R_wp_) values for each refinement are plotted as a function of the refinement range for the variable range refinement and as a function of the high-*r* edge of the box (i.e. x = 15 for the 5–10 Å box) for the box-car method. For each method insets are provided to show an example of a characteristic refinement.

From the variable range refinement method, at both temperatures the *R*3*m* fit yields the lowest R_wp_ for the 1–5 Å and 1–10 Å refinements where the local Ti-O distortions dominate the fit quality. At larger length scales the *Amm*2 (for 225 K) and *P*4*mm* (for 290 K) average structures yield the best fits for as the penalty from the poorly fit local Ti-O peaks are overcome by a better overall fit to the high-*r* data. The comparatively larger R_wp_ values in the first box from 1 to 5 Å for each data set are due to the larger influence of low-*r* noise on the refinement.

For the box-car refinements at 225 K, the rhombohedral *R*3*m* space group fits the data better until the 20–30 Å box where the *Amm*2 space group yields a lower R_wp_. However the next two refinement boxes, 25–35 and 30–40 Å, are best fit by the *R*3*m* structure with anomalously low R_wp_ values. At higher r-ranges the *Amm*2 average structure again yields the lower R_wp_. These results are unclear and the back-switching of the fit criterion is likely due to the decreased intensity of the PDF signal at high *r*-ranges. Further information on the 20–30, 25–35, and 30–40 Å box-car refinements are presented in the supplementary information.

The box-car method refinements at 290 K are more clear where the local *R*3*m* structure fits the data better for the first 1–10 Å box that incorporates the local Ti-O_6_ distortion. At higher *r*-ranges the *P*4*mm* structure yields the lower R_wp_ as expected from the average structure refinements. From these results, the coherence length of the local rhombohedral distortions can be estimated to be around ~10 Å at 290 K. At larger distances, the distortions are misaligned relative to one another resulting in the *P*4*mm* average structure yielding the superior fit. This observed phenomenon is formalized as the order-disorder model for BaTiO_3_. An example of the order-disorder model is well visualized in Fig. 3 within the work by Senn *et al.*^16^.

## Discussion

For MgTiO_3_, CaTiO_3_, and SrTiO_3_ the refinements average and local structures were internally consistent at each temperature. However the average structures for BaTiO_3_ (*Amm*2 at 225 and *P*4*mm* at 290 K) show distinct features with a different symmetry than the symmetry observed at the local structure. There are clear rhombohedral distortions present in the PDF data at both temperatures which are consistent with the order-disorder model for BaTiO_3_. The local titanium octahedra distortions are only observed in the first Ti-O correlations around 2 Å. Furthermore, these distortions average out above 10 Å in the PDF data and are not observable in the neutron diffraction data.

The contrast in local structure versus long range average structure observed in BaTiO_3_ has profound impacts on the physical properties of the material. The other compounds in this study, MgTiO_3_, CaTiO_3_, SrTiO_3_, show a clear correlation between the local and average structure and all exhibit a linear dielectric response. In contrast, in BaTiO_3_ the unique structural features detailed in this work are linked to its strong non-linear characteristics which are observed in physical phenomena in which there is a correlation between the local structure and the long-range structure. One example includes the occurrence of ferroelectricity in BaTiO_3_, in which the local dipole moments interact to form a spontaneous polarization. Another consequence of the variable local structural distortions in BaTiO_3_ is the resultant flat potential energy landscape that leads to the high polarizability and ease of switching of the Ti^4+^ ion^23^. Macroscopically, this is observed in BaTiO_3_ ceramics as large relative permittivities, large polarizations, and large field induced electromechanical strains. This is also clearly relevant to other systems where these contrasting structural length scales influence the material response such as in relaxor ferroelectrics^24^. Thus, materials systems in which there is a disconnect between the long-range average structure and local structural arrangements are of both scientific and technological interest.

In this work, we present the average and local structure of group II titanates, ATiO_3_ (A^2+^ = Mg, Ca, Sr, Ba). For all materials except BaTiO_3_, there are no observed differences between the local and average structures. It was shown that MgTiO_3_ crystallizes into the ilmenite structure with trigonal *R*$\bar{3}$ symmetry. Both CaTiO_3_ and SrTiO_3_ crystallize in the perovskite structure with orthorhombic *Pbnm* and cubic *Pm*$\bar{3}$*m* symmetry, respectively. The average structure of BaTiO_3_ at 225 K is orthorhombic *Amm*2 and tetragonal *P*4*mm* at 290 K. However, the local structure at both temperatures is best described with small box modeling as rhombohedral *R*3*m* when the refinements were confined to data below ~10 Å, showing that the coherence length of the distortions is limited to only one to two unit cells in the structure. At larger length scales, the PDF data statistically fits the average structural model better, as the vector sum of the local titanium displacement appears to be in the [011] direction at 225 K and the [001] direction at 290 K corresponding to the orthorhombic *Amm*2 and tetragonal *P*4*mm* average structures, respectively.

## Methods

Polycrystalline samples of MgTiO_3_, CaTiO_3_, SrTiO_3_, and BaTiO_3_ were prepared via standard solid-state synthesis. For each compound, stoichiometric amounts of the component oxide and carbonate reagents were mixed. The reagents MgO (Alfa Aesar 99.95%), CaCO_3_ (Alfa Aesar 99.997%), SrCO_3_ (Alfa Aesar 99.994%), BaCO_3_ (Strem Chemicals Inc. 99.9%), and TiO_2_ (Sigma Aldrich 99.997%) were used. The carbonate powders (CaCO_3_, SrCO_3_, and BaCO_3_) were heated at 110 °C for 24 h before being weighed. After the initial mixing of powder reagents the mixtures were ground well with a mortar and pestle. The homogenized samples were then ball milled for 4 h in a planetary micromill (Pullverisette 7 Classic Line, Fritsch). For the milling process, each sample mixture was placed in a sealed container with 20 mL of ethanol and eight 10 mm yttria-stabilized zirconia (YSZ) balls. The milled solutions were dried in air at room temperature. Then the dried powders were calcined in alumina crucibles at 800 °C for 24 h, 900 °C for 6 h, 700 °C for 4 h, and 1100 °C for 4 h for MgTiO_3_, CaTiO_3_, SrTiO_3_, and BaTiO_3_ respectively, with a 4 h step at 450 °C (to allow for degassing of carbonates). The calcined powders were ground well and pressed, uniaxially in a 10-mm die (Carver Inc.) at 2 tons for 2 mins, into pellets. The sample pellets were placed in closed alumina crucibles and sintered at 1200 °C for 12 h, 1350 °C for 12 h, 1100 °C for 12 h, and 1250 °C for 6 h for MgTiO_3_, CaTiO_3_, SrTiO_3_, and BaTiO_3_ respectively. A heating and cooling rate of 5 °C/min was used for the calcination and sintering steps for all samples. The sintered pellets were ground well and loaded into X-ray diffraction sample holders. X-ray diffraction data was collected using a benchtop X-ray diffractometer (Miniflex 600, Rigaku) to determine the phase purity.

To collect neutron total scattering measurements, samples were loaded in 2 mm diameter quartz capillaries (Hampton) and sent to the Nanoscale Ordered Materials Diffractometer (NOMAD)^25^ beam-line at the Spallation Neutron Source (SNS)^26^ at Oak Ridge National Labs (ORNL). Data were collected for each sample at 225 and 290 K with a collection time of 30 mins and a heating ramp rate of 60 K/min.

To determine the average structure, Pawley fitting^27^ was performed on the collected neutron total scattering data using the Topas Academic (Version 6) software package^28,29^. The starting models for each refinement were taken from the literature^6,11,20,30^. For each Pawley fit the background terms, unit cell parameters, and peak profiles were refined. The background for each refinement was modelled with a 10 term polynomial. The results of the Pawley fits were used as starting models for Rietveld analysis^31,32^ (also performed with Topas) where the scale factor, atomic positions and atomic displacement parameters were refined.

To investigate the local structure of these materials the total scattering data were transformed to the pair distribution function (PDF), *G*(*r*), via a sine Fourier transform^33^. This transformation was performed with in-house software at ORNL with *Q*_min_ of 0.50 Å^−1^ and *Q*_max_ of 30.00 Å^−1^. Again Topas (Version 6) was used to perform small-box modeling using the refined average structure as starting models. The scale, lattice parameters, atomic displacement parameters, and atomic positions were refined. The delta2, *Q*_damp_, and *Q*_broad_ parameters were refined from both a nickel and a silicon standard.

## Supplementary information

Supplementary Information.
