# Supplementary material for: Neutron Total Scattering Studies of Group II Titanates (ATiO3, A2+ = Mg, Ca, Sr, Ba)
Source: Sci Rep. 2020 Feb 28;10:3729. doi: 10.1038/s41598-020-60475-8 (PMC7048731; doi:10.1038/s41598-020-60475-8)
Supplement: Supplementary file 1 — Supplementary Information. [file 41598_2020_60475_MOESM1_ESM.docx]

Neutron Total Scattering Studies of Group II Titanates (ATiO_3_, A^2+^ = Mg, Ca, Sr, Ba)

Charles M. Culbertson, Alexander T. Flak, Michael Yatskin, Paul H.-Y. Cheong, David P. Cann, and Michelle R. Dolgos

**Supplementary Note 1**

The phase pure diffractograms from a bench-top X-ray diffractometer (Miniflex 600) for each compound are shown in Fig. S1a-d. There was a Tungsten L-α reflection observed in all samples, denoted by †. Furthermore, there was a minor MgTi_2_O_5_ impurity phase denoted by * in Fig. S1a.

**
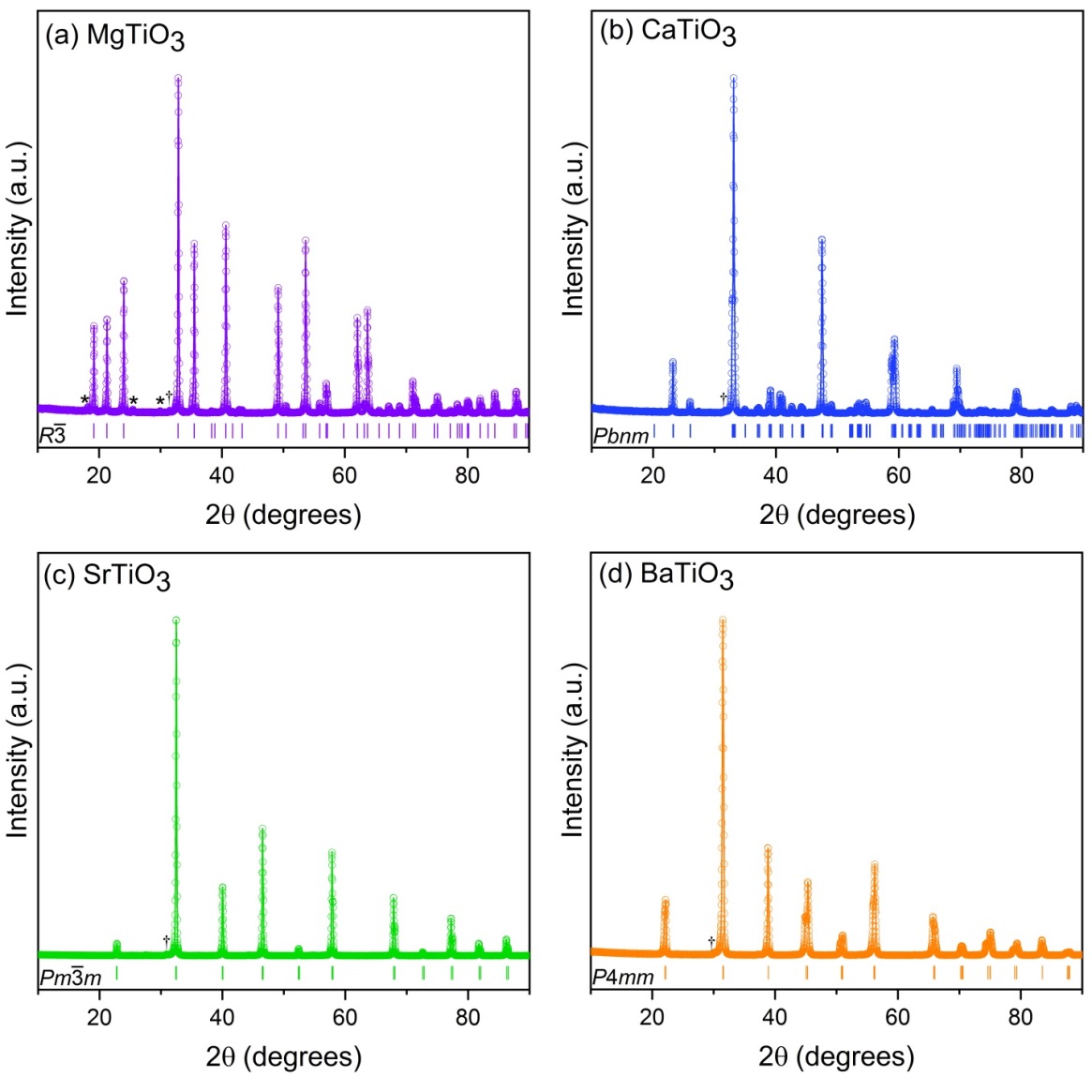
**

Supplementary Figure S1. Benchtop X-ray diffraction results for (a) MgTiO_3_, (b) CaTiO_3_, (c) SrTiO_3_, and (d) BaTiO_3_, with *hkl* ticks shown below with the corresponding space group labels. *MgTi_2_O_5_ impurity phase. †Tungsten L-α reflection as determined from a Silicon standard.

**Supplementary Note 2**

For MgTiO_3_ the data at 225 and 290 K was well modelled in the trigonal *R*$\bar{3}$ space group. The results at 225 K are shown in Figure S2 and tabulated in Table S1. The tabulated results for MgTiO_3_ at 290 K are shown in Table S2.


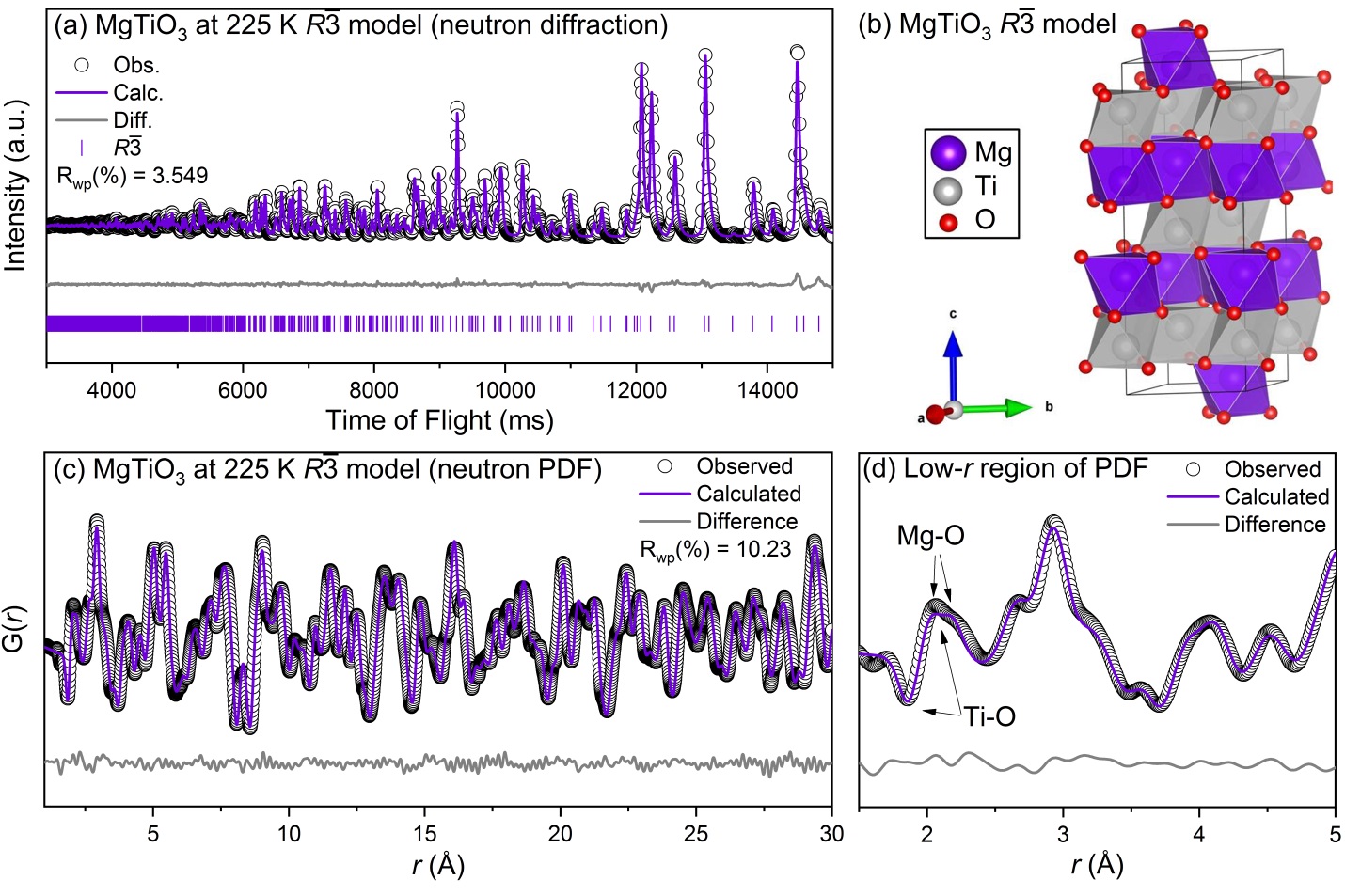


Supplementary Figure S2. (a) Rietveld refinement of neutron diffraction, (b) refined model, (c) small-box modeling of neutron PDF, and (d) zoom-in of neutron PDF for MgTiO_3_ at 225 K with the *R*$\bar{3}$ space group. Data (identified by black circles) and refined models (continuous lines) are shown, along with the difference pattern and *hkl* indices below (diffraction data only).

Supplementary Table S1. Rietveld and neutron PDF (1-30 Å) refinement parameters for MgTiO_3_ at 225 K with the *R*$\bar{3}$ space group.

| MgTiO_3_ at 225 K | | | | | | | | | | | |
| --- | --- | --- | --- | --- | --- | --- | --- | --- | --- | --- | --- |
| Neutron Diffraction Refinement: | | | | | | Neutron PDF Refinement: | | | | | |
| a (Å) | 5.0554(1) | α (°) | 90 | R_p_ | 2.688 | a (Å) | 5.0571(6) | α (°) | 90 |  |  |
| b (Å) | 5.0554(1) | β (°) | 90 | R_wp_ | 3.549 | b (Å) | 5.0571(6) | β (°) | 90 | R_wp_ | 10.226 |
| c (Å) | 13.8966(1) | γ (°) | 120 | red. χ^2^ | 2.010 | c (Å) | 13.9031(1) | γ (°) | 120 | red. χ^2^ | 0.1774 |
| Atomic Position | x | y | z | Occ. | Beq | Atomic Position | x | y | z | Occ. | Beq |
| Mg | 0 | 0 | 0.3554(5) | 1 | 0.383(1) | Mg | 0 | 0 | 0.355(9) | 1 | 0.43(6) |
| Ti | 0 | 0 | 0.1448(7) | 1 | 0.580(5) | Ti | 0 | 0 | 0.144(8) | 1 | 0.35(4) |
| O | 0.3159(6) | 0.0214(4) | 0.2463(9) | 1 | 0.485(1) | O | 0.315(9) | 0.020(9) | 0.246(6) | 1 | 0.44(3) |

Supplementary Table S2. Rietveld and neutron PDF (1-30 Å) refinement parameters for MgTiO_3_ at 290 K with the *R*$\bar{3}$ space group.

| MgTiO_3_ at 290 K | | | | | | | | | | | |
| --- | --- | --- | --- | --- | --- | --- | --- | --- | --- | --- | --- |
| Neutron Diffraction Refinement: | | | | | | Neutron PDF Refinement: | | | | | |
| a (Å) | 5.0574(2) | α (°) | 90 | R_p_ | 3.208 | a (Å) | 5.0591(2) | α (°) | 90 |  |  |
| b (Å) | 5.0574(2) | β (°) | 90 | R_wp_ | 4.146 | b (Å) | 5.0591(2) | β (°) | 90 | R_wp_ | 10.012 |
| c (Å) | 13.9044(3) | γ (°) | 120 | red. χ^2^ | 2.027 | c (Å) | 13.9118(6) | γ (°) | 120 | red. χ^2^ | 0.2022 |
| Atomic Position | x | y | z | Occ. | Beq | Atomic Position | x | y | z | Occ. | Beq |
| Mg | 0 | 0 | 0.3554(2) | 1 | 0.430(2) | Mg | 0 | 0 | 0.356(1) | 1 | 0.47(6) |
| Ti | 0 | 0 | 0.1448(1) | 1 | 0.639(3) | Ti | 0 | 0 | 0.144(9) | 1 | 0.39(8) |
| O | 0.3159(6) | 0.0215(5) | 0.2463(4) | 1 | 0.543(1) | O | 0.316(2) | 0.021(2) | 0.246(6) | 1 | 0.49(1) |

**Supplementary Note 3**

For CaTiO_3_ the data at 225 and 290 K was well modelled in the orthorhombic *Pbnm* space group. The results at 225 K are shown in Figure S3 and tabulated in Table S3. The tabulated results for CaTiO_3_ at 290 K are shown in Table S4.


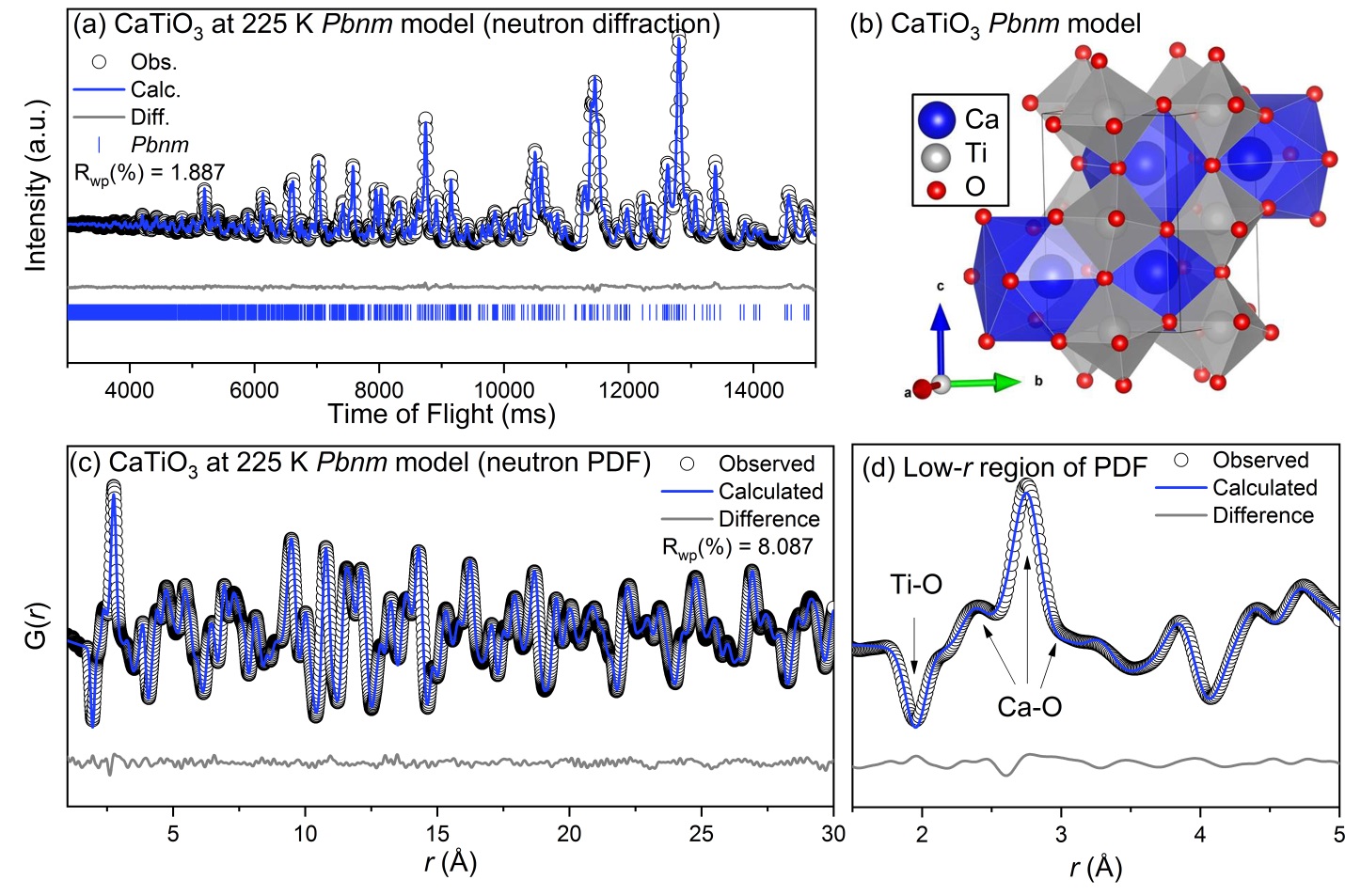


Supplementary Figure S3. (a) Rietveld refinement of neutron diffraction, (b) refined model, (c) small-box modeling of neutron PDF, and (d) zoom-in of neutron PDF for CaTiO_3_ at 225 K with the *Pbnm* space group. Data (identified by black circles) and refined models (continuous lines) are shown, along with the difference pattern and *hkl* indices below (diffraction data only).

Supplementary Table S3. Rietveld and neutron PDF (1-30 Å) refinement parameters for CaTiO_3_ at 225 K with the orthorhombic *Pbnm* space group.

| CaTiO_3_ at 225 K | | | | | | | | | | | |
| --- | --- | --- | --- | --- | --- | --- | --- | --- | --- | --- | --- |
| Neutron Diffraction Refinement: | | | | | | Neutron PDF Refinement: | | | | | |
| a (Å) | 5.3774(5) | α (°) | 90 | R_p_ | 1.438 | a (Å) | 5.3769(7) | α (°) | 90 |  |  |
| b (Å) | 5.4424(3) | β (°) | 90 | R_wp_ | 1.887 | b (Å) | 5.4440(5) | β (°) | 90 | R_wp_ | 8.087 |
| c (Å) | 7.6385(6) | γ (°) | 90 | red. χ^2^ | 2.236 | c (Å) | 7.6438(3) | γ (°) | 90 | red. χ^2^ | 0.1235 |
| Atomic Position | x | y | z | Occ. | Beq | Atomic Position | x | y | z | Occ. | Beq |
| Ca | 0.9932(1) | 0.0361(1) | 0.25 | 1 | 0.565(2) | Ca | 0.992(1) | 0.036(2) | 0.25 | 1 | 0.48(6) |
| Ti | 0 | 0.5 | 0 | 1 | 0.386(9) | Ti | 0 | 0.5 | 0 | 1 | 0.33(3) |
| O1 | 0.0729(6) | 0.4831(2) | 0.25 | 1 | 0.451(1) | O1 | 0.072(5) | 0.482(1) | 0.25 | 1 | 0.39(1) |
| O2 | 0.7109(7) | 0.2892(2) | 0.0378(3) | 1 | 0.482(4) | O2 | 0.711(3) | 0.288(6) | 0.038(4) | 1 | 0.44(3) |

Supplementary Table S4. Rietveld and neutron PDF (1-30 Å) refinement parameters for CaTiO_3_ at 290 K with the orthorhombic *Pbnm* space group.

| CaTiO_3_ at 290 K | | | | | | | | | | | |
| --- | --- | --- | --- | --- | --- | --- | --- | --- | --- | --- | --- |
| Neutron Diffraction Refinement: | | | | | | Neutron PDF Refinement: | | | | | |
| a (Å) | 5.3822(5) | α (°) | 90 | R_p_ | 2.187 | a (Å) | 5.3814(8) | α (°) | 90 |  |  |
| b (Å) | 5.4437(7) | β (°) | 90 | R_wp_ | 3.108 | b (Å) | 5.4456(3) | β (°) | 90 | R_wp_ | 8.051 |
| c (Å) | 7.6438(5) | γ (°) | 90 | red. χ^2^ | 3.399 | c (Å) | 7.6491(5) | γ (°) | 90 | red. χ^2^ | 0.1263 |
| Atomic Position | x | y | z | Occ. | Beq | Atomic Position | x | y | z | Occ. | Beq |
| Ca | 0.9961(4) | 0.0360(8) | 0.25 | 1 | 0.689(2) | Ca | 0.992(6) | 0.035(3) | 0.25 | 1 | 0.59(5) |
| Ti | 0 | 0.5 | 0 | 1 | 0.447(4) | Ti | 0 | 0.5 | 0 | 1 | 0.37(5) |
| O1 | 0.0728(3) | 0.4829(8) | 0.25 | 1 | 0.497(7) | O1 | 0.072(1) | 0.482(1) | 0.25 | 1 | 0.42(2) |
| O2 | 0.7112(8) | 0.2895(6) | 0.0371(5) | 1 | 0.481(2) | O2 | 0.711(9) | 0.288(1) | 0.038(2) | 1 | 0.50(1) |

**Supplementary Note 4**

For SrTiO_3_ the data at 225 and 290 K was well modelled in the cubic *Pm*$\bar{3}$*m* space group. The results at 225 K are shown in Figure S4 and tabulated in Table S5. The tabulated results for SrTiO_3_ at 290 K are shown in Table S6.

**
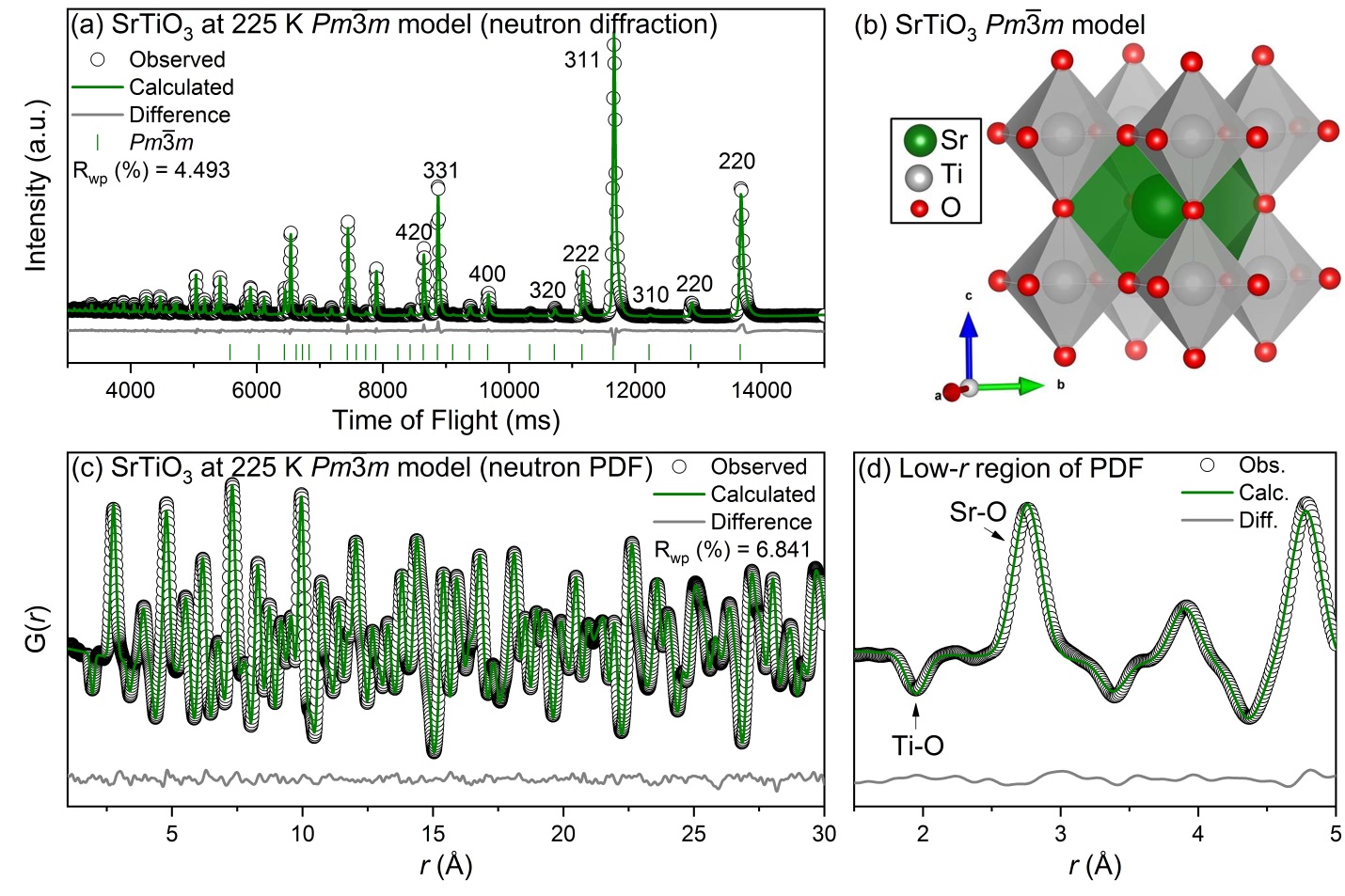
**

Supplementary Figure S4. (a) Rietveld refinement of neutron diffraction, (b) refined model, (c) small-box modeling of neutron PDF, and (d) zoom-in of neutron PDF for SrTiO_3_ at 225 K with the *Pm*$\bar{3}$*m* space group. Data (identified by black circles) and refined models (continuous lines) are shown, along with the difference pattern and *hkl* indices below (diffraction data only).

Supplementary Table S5. Rietveld and neutron PDF (1-30 Å) refinement parameters for SrTiO_3_ at 225 K with the cubic *Pm*$\bar{3}$*m* space group.

| SrTiO_3_ at 225 K | | | | | | | | | | | |
| --- | --- | --- | --- | --- | --- | --- | --- | --- | --- | --- | --- |
| Neutron Diffraction Refinement: *Pm*$\bar{3}$*m* | | | | | | Neutron PDF Refinement: *Pm*$\bar{3}$*m* | | | | | |
| a (Å) | 3.9046(4) | α (°) | 90 | R_p_ | 3.572 | a (Å) | 3.9064(2) | α (°) | 90 |  |  |
| b (Å) | 3.9046(4) | β (°) | 90 | R_wp_ | 4.493 | b (Å) | 3.9064(2) | β (°) | 90 | R_wp_ | 6.841 |
| c (Å) | 3.9046(4) | γ (°) | 90 | red. χ^2^ | 4.028 | c (Å) | 3.9064(2) | γ (°) | 90 | red. χ^2^ | 0.2268 |
| Atomic Position | x | y | z | Occ. | Beq | Atomic Position | x | y | z | Occ. | Beq |
| Sr | 0 | 0 | 0 | 1 | 0.408(3) | Sr | 0 | 0 | 0 | 1 | 0.44(6) |
| Ti | 0.5 | 0.5 | 0.5 | 1 | 0.332(4) | Ti | 0.5 | 0.5 | 0.5 | 1 | 0.32(1) |
| O | 0.5 | 0.5 | 0 | 1 | 0.555(2) | O | 0.5 | 0.5 | 0 | 1 | 0.54(5) |

Supplementary Table S6. Rietveld and neutron PDF (1-30 Å) refinement parameters for SrTiO_3_ at 290 K with the cubic *Pm*$\bar{3}$*m* space group.

| SrTiO_3_ at 290 K | | | | | | | | | | | |
| --- | --- | --- | --- | --- | --- | --- | --- | --- | --- | --- | --- |
| Neutron Diffraction Refinement: *Pm*$\bar{3}$*m* | | | | | | Neutron PDF Refinement: *Pm*$\bar{3}$*m* | | | | | |
| a (Å) | 3.9067(9) | α (°) | 90 | R_p_ | 3.481 | a (Å) | 3.9085(8) | α (°) | 90 |  |  |
| b (Å) | 3.9067(9) | β (°) | 90 | R_wp_ | 4.309 | b (Å) | 3.9085(8) | β (°) | 90 | R_wp_ | 6.969 |
| c (Å) | 3.9067(9) | γ (°) | 90 | red. χ^2^ | 3.548 | c (Å) | 3.9085(8) | γ (°) | 90 | red. χ^2^ | 0.2386 |
| Atomic Position | x | y | z | Occ. | Beq | Atomic Position | x | y | z | Occ. | Beq |
| Sr | 0 | 0 | 0 | 1 | 0.506(4) | Sr | 0 | 0 | 0 | 1 | 0.52(4) |
| Ti | 0.5 | 0.5 | 0.5 | 1 | 0.396(1) | Ti | 0.5 | 0.5 | 0.5 | 1 | 0.37(5) |
| O | 0.5 | 0.5 | 0 | 1 | 0.637(8) | O | 0.5 | 0.5 | 0 | 1 | 0.62(7) |

**Supplementary Note 5**

For BaTiO_3_ (BT) the tabulated results for the refinements at 225 K in the orthorhombic *Amm*2 space group are show in Table S7. Similarly, the tabulated results for the refinements at 290 K in the tetragonal *P*4*mm* space group are shown in Table S8. The tabulated results from the local structure refinement (from 1-10 Å) at 290 K in the rhombohedral *R*3*m* space group are shown in table S9. Furthermore, the results of the box-car refinements are shown in figure S5.

Supplementary Table S7. Rietveld and neutron PDF (1-30 Å) refinement parameters for BaTiO_3_ at 225 K.

| BaTiO_3_ at 225 K | | | | | | | | | | | |
| --- | --- | --- | --- | --- | --- | --- | --- | --- | --- | --- | --- |
| Neutron Diffraction Refinement: *Amm*2 | | | | | | Neutron PDF Refinement: *Amm*2 | | | | | |
| a (Å) | 3.9886(4) | α (°) | 90 | R_p_ | 2.327 | a (Å) | 3.991(1) | α (°) | 90 |  |  |
| b (Å) | 5.6749(3) | β (°) | 90 | R_wp_ | 3.421 | b (Å) | 5.678(9) | β (°) | 90 | R_wp_ | 6.454 |
| c (Å) | 5.6926(1) | γ (°) | 90 | red. χ^2^ | 5.057 | c (Å) | 5.694(4) | γ (°) | 90 | red. χ^2^ | 0.2026 |
| Atomic Position | x | y | z | Occ. | Beq | Atomic Position | x | y | z | Occ. | Beq |
| Ba | 0 | 0 | 0 | 1 | 0.292(7) | Ba | 0 | 0 | 0 | 1 | 0.38(5) |
| Ti | 0.5 | 0 | 0.511(3) | 1 | 0.596(5) | Ti | 0.5 | 0 | 0.514(1) | 1 | 0.29(5) |
| O1 | 0 | 0 | 0.487(1) | 1 | 0.341(4) | O1 | 0 | 0 | 0.487(1) | 1 | 0.31(2) |
| O2 | 0.5 | 0.255(4) | 0.233(8) | 1 | 0.516(1) | O2 | 0.5 | 0.253(5) | 0.231(7) | 1 | 0.46(8) |

Supplementary Table S8. Structural parameters from the neutron PDF (1-10 Å) refinement for BaTiO_3_ at 225 K.

| BaTiO_3_ at 225 K Neutron PDF Refinement: *R*3*m* | | | | | |
| --- | --- | --- | --- | --- | --- |
| a (Å) | 4.0141(8) | α (°) | 89.63(6) |  |  |
| b (Å) | 4.0141(8) | β (°) | 89.63(6) | R_wp_ | 4.203 |
| c (Å) | 4.0141(8) | γ (°) | 89.63(6) | red. χ^2^ | 0.1463 |
| Atomic Position | x | y | z | Occ. | Beq |
| Ba | 0.012(1) | 0.012(1) | 0.012(1) | 1 | 0.28(3) |
| Ti | 0.5 | 0.5 | 0.5 | 1 | 0.36(2) |
| O | 0.523(2) | 0.523(2) | 0.029(2) | 1 | 0.40(1) |

Supplementary Table S9. Rietveld and neutron PDF (1-30 Å) refinement parameters for BaTiO_3_ at 290 K.

| BaTiO_3_ at 290 K | | | | | | | | | | | |
| --- | --- | --- | --- | --- | --- | --- | --- | --- | --- | --- | --- |
| Neutron Diffraction Refinement: *P*4*mm* | | | | | | Neutron PDF Refinement: *P*4*mm* | | | | | |
| a (Å) | 3.9976(8) | α (°) | 90 | R_p_ | 3.604 | a (Å) | 3.999(4) | α (°) | 90 |  |  |
| b (Å) | 3.9976(8) | β (°) | 90 | R_wp_ | 4.841 | b (Å) | 3.999(4) | β (°) | 90 | R_wp_ | 6.714 |
| c (Å) | 4.0347(6) | γ (°) | 90 | red. χ^2^ | 6.754 | c (Å) | 4.038(9) | γ (°) | 90 | red. χ^2^ | 0.2087 |
| Atomic Position | x | y | z | Occ. | Beq | Atomic Position | x | y | z | Occ. | Beq |
| Ba | 0 | 0 | 0 | 1 | 0.029(6) | Ba | 0 | 0 | 0 | 1 | 0.36(3) |
| Ti | 0.5 | 0.5 | 0.508(5) | 1 | 1.08(4) | Ti | 0.5 | 0.5 | 0.514(8) | 1 | 0.51(6) |
| O1 | 0.5 | 0.5 | 0.023(3) | 1 | 1.22(1) | O1 | 0.5 | 0.5 | -0.028(4) | 1 | 0.59(3) |
| O2 | 0.5 | 0 | 0.506(1) | 1 | 0.481(6) | O2 | 0.5 | 0 | 0.485(8) | 1 | 0.46(1) |

Supplementary Table S10. Structural parameters from the neutron PDF (1-10 Å) refinement for BaTiO_3_ at 290 K.

| BaTiO_3_ at 290 K Neutron PDF Refinement: *R*3*m* | | | | | |
| --- | --- | --- | --- | --- | --- |
| a (Å) | 4.0155(8) | α (°) | 89.58(4) |  |  |
| b (Å) | 4.0155(8) | β (°) | 89.58(4) | R_wp_ | 4.349 |
| c (Å) | 4.0155(8) | γ (°) | 89.58(4) | red. χ^2^ | 0.1504 |
| Atomic Position | x | y | z | Occ. | Beq |
| Ba | 0.009(9) | 0.009(9) | 0.009(9) | 1 | 0.35(3) |
| Ti | 0.5 | 0.5 | 0.5 | 1 | 0.48(8) |
| O | 0.521(7) | 0.521(7) | 0.027(2) | 1 | 0.44(9) |

**Supplementary Note 6**

The box-car refinement results for BaTiO_­3_ at 225 K for the 20-30, 25-35, and 30-40 Å boxes is shown in supplementary figure S5 with the rhombohedral *R*3*m* and orthorhombic *Amm*2 fits. As mentioned in the main text, at low-*r* the rhombohedral model fit best, then at intermediate distances the orthorhombic model fits best. Surprisingly though for the 25-35 and 30-40 Å boxes the rhombohedral model fit significantly better. Visually it is difficult to determine a significant difference between the fits, but in general the orthorhombic model provides sharper features that are not observed and thus yields a poorer fit. At higher-r ranges, the average orthorhombic structure yields the better fit as expected.


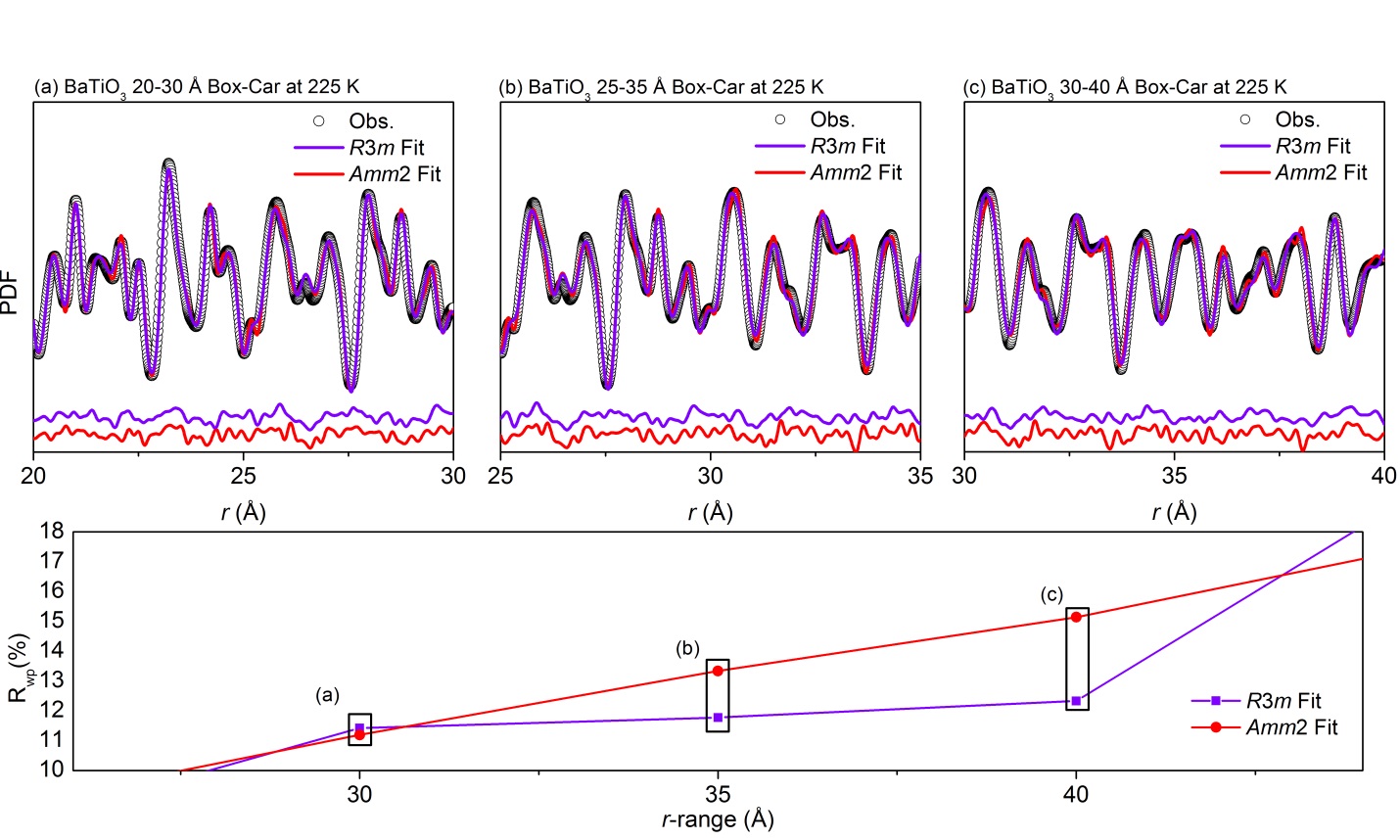


Supplementary Figure S5. Box-car refinement results (below) and fits (above) for BaTiO_3_ at 225 K for the (a) 20-30, (b) 25-35, and (c) 30-40 Å boxes.
